# Mapping parents’ journey following prenatal diagnosis of CHD: a qualitative study

**DOI:** 10.1017/S1047951122002505

**Published:** 2022-08-09

**Authors:** Kelly W. Harris, Catherine M. Hammack-Aviran, Kathleen M. Brelsford, Ann Kavanaugh-McHugh, Ellen Wright Clayton

**Affiliations:** 1 Division of General Pediatrics, Vanderbilt University Medical Center, Nashville, TN, USA; 2 Center for Biomedical Ethics and Society, Vanderbilt University Medical Center, Nashville, TN, USA; 3 Section of Palliative Care and Medical Ethics, University of Pittsburgh Medical Center, Pittsburgh, PA, USA; 4 Division of Pediatric Cardiology, Department of Pediatrics, Vanderbilt University Medical Center, Nashville, TN, USA; 5 School of Law, Vanderbilt University, Nashville, TN, USA

**Keywords:** CHD, qualitative research, counselling, prenatal diagnosis

## Abstract

**Objective:**

To better understand parents’ accounts of their prenatal and postnatal experience after prenatal diagnosis of CHD – particularly emotional processing and coping mechanisms – to identify strategies to improve support.

**Methods:**

This single-centre, longitudinal qualitative study included pregnant mothers and their support persons seen in Fetal Cardiology Clinic at Vanderbilt Children’s Hospital from May through August 2019 for probable complex CHD. Twenty-seven individuals from 17 families participated in 62 phone interviews during pregnancy and postpartum: 27 conducted after the initial prenatal cardiology consultation, 15 after a follow-up prenatal visit, and 20 after birth. Applied thematic analysis approach was used to code and analyse transcribed interviews. Coding and codebook revisions occurred iteratively; intercoder reliability was >80%.

**Results:**

Patients included mothers (16 [59%]), fathers (8 [30%]), and other support persons (3 [11%]). Initial fetal diagnoses included a range of moderate to severe CHD. Prenatally, parents sought to maintain hope while understanding the diagnosis; planning for the future rather than focusing on day-to-day was more common if prognoses were better. Postnatally, with confirmation of prenatal diagnoses, parents’ sense of control expanded, and they desired more active engagement in clinical decision making.

**Conclusions:**

To enhance effective communication and support, understanding how parents conceptualise hope in relation to diagnosis and how that may evolve overtime is critical. Expectant parents whose child has a significant risk of mortality may demonstrate hope by focusing on positivity. As prognostic uncertainty diminishes postpartum, the parental role on the team may shift, requiring clinicians to provide different support.

CHD is diagnosed in approximately 1% of births in the United States of America; a substantial and increasing proportion of these diagnoses is made prenatally.^1,2^ Physicians may assume that prenatal diagnosis is beneficial, allowing parents additional emotional processing and decision making about continuing pregnancy and intended delivery location.^3–8^ Prenatal diagnosis does lead to improved parental understanding of CHD at the time of Neonatal ICU (NICU) discharge.^9^ However, studies have demonstrated that receiving a prenatal diagnosis of CHD, on average, elicits greater parental psychological stress, anxiety, and depressive symptoms at hospital discharge and for months after birth when compared with receiving a postnatal diagnosis.^10–20^ Researchers have called for longitudinal studies of parents to begin to understand this phenomenon.^14,21–26^ Prior qualitative studies have begun to investigate the experience of mothers after receiving a prenatal diagnosis;^21,27–35^ this study extends that knowledge by tracking parents’ experience over time from prenatal diagnosis until after birth.

The first publication from this study focused on the prenatal parental experience after CHD diagnosis, especially sources of stress, and identified uncertainty as a pervasive theme, regarding both concrete questions about scheduling, logistics or next steps, and long-term unknown variables.^36^ In order to identify interventions to improve parental support, we sought to understand how the parental experience, especially emotional processing and coping mechanisms, evolves over time from prenatal diagnosis through the postpartum period. This study sheds light on what may be occurring when parents talk about having hope, as distinct from denial, wishes, or naïve optimism, in order to help clinicians to respond to and provide support for families more effectively.

## Methods

### Study design and setting

We conducted a single-centre, longitudinal, qualitative study of pregnant mothers and their partners or support persons seen in Fetal Cardiology Clinic at Vanderbilt Children’s Hospital for probable complex CHD.^36^ Patients were enrolled prior to the pregnant mother’s first consultation appointment. With the consent of the fetal cardiologists and the parents, in-clinic prenatal counseling was observed at the initial prenatal visit and one follow-up visit.

One investigator (KWH) conducted audio-recorded semi-structured interviews with each participant at three time points, one after each observed prenatal visit and one postnatally. Written consent was obtained from all patients at enrollment, and verbal permission prior to each interview. The average interview length was 27 minutes. This study was approved by the Vanderbilt University Institutional Review Board.

### Data collection

**Figure 1.**
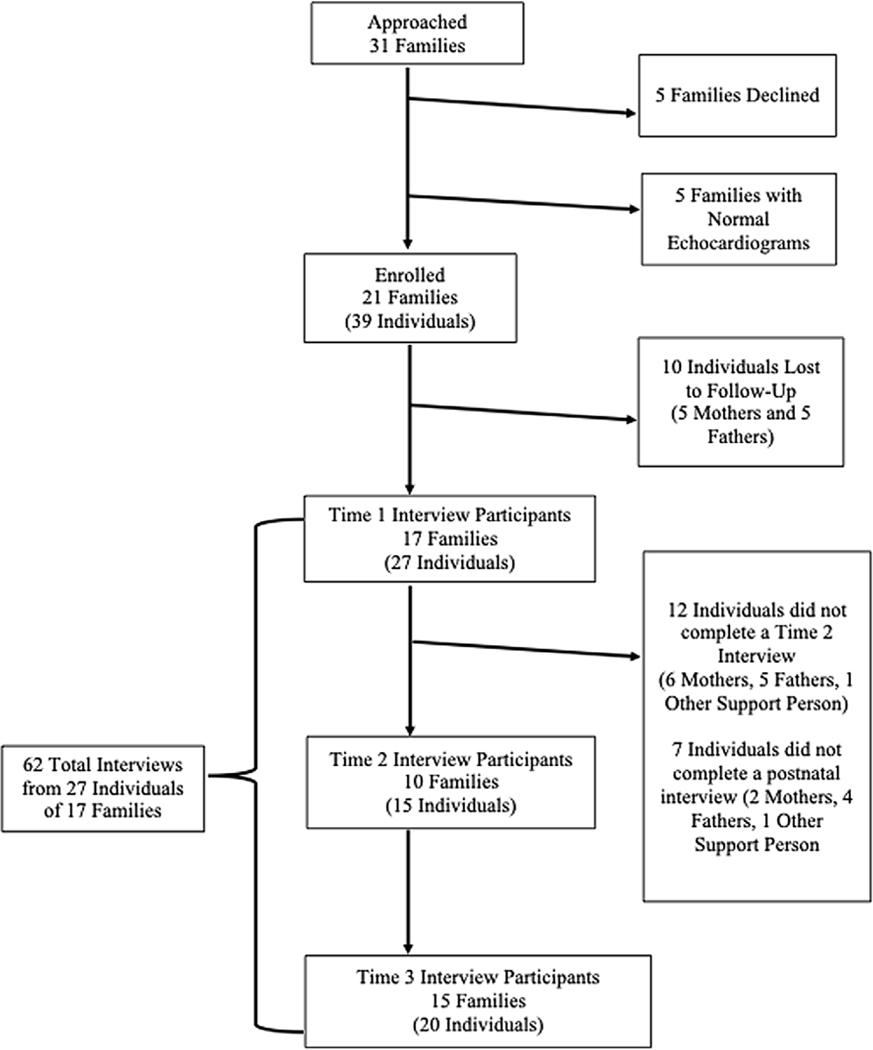
Study cohort flowchart, including initial enrollment and participation in semistructured phone interviews over time.

Patients were enrolled from May to August, 2019; 31 families were approached, 5 families declined enrollment; the participation rate was 84%. Five families were excluded following normal echocardiograms. Of the 37 individuals from 21 families who enrolled and were eligible, 10 were lost to follow-up prior to being interviewed. Active patients included 27 individuals from 17 families; 62 interviews were conducted from May, 2019 to January 2020 (see Fig 1). The median timing for prenatal interviews was 13 days after a fetal cardiology clinic visit, and for postnatal interviews, 30 days after birth.

A semi-structured interview guide with 13 primary questions explored patients’ overall experience, anticipation of the future, factors influencing their experience, and feelings of empowerment (see Appendix). This paper reports responses to questions about emotional processing, coping, and support. Nearly all interviews were conducted on the phone with individual patients; one interview was conducted in-person and with both a mother and father present, per their request.

### Data analysis

Interview transcripts were analysed using NVivo 12 (QSR International). An iterative process was used to code and analyse data using an applied thematic analysis approach, following the Consolidated Criteria for Reporting Qualitative Research reporting guidelines for qualitative studies.^37^ One author (KWH) developed an initial structural and content codebook including code definitions, inclusion and exclusion criteria, and illustrative examples.^38^ Two authors (KWH and KMB) systematically refined the codebook through an iterative process of application, discussion, and revision. KWH was the primary coder for all transcripts. KMB and CMHA each independently reviewed one-fifth of transcripts at fixed intervals to maintain >80% interrater reliability. Discrepancies in coding were resolved through discussion until consensus was reached. Post hoc, themes were compared across time (prenatal and postnatal interviews) and diagnosis (higher and lower risk of mortality).

Participant quotes are identified first by family number (1–21), then by participant role (M = mother, F = father, O = any other support person), time point of interview (1–3; 1 and 2 occurred prenatally and 3 occurred postnatally), and finally by risk of mortality (H = higher, L = lower). Thus, 5.F.2.H indicates a quote from a father of family number 5 during the second interview with a higher risk of mortality diagnosis.

## Results

### Patient characteristics

Patients represented a range of demographic characteristics (Table 1). Most interviewees were mothers (16 [59%]) or fathers (8 [30%]). The majority self-identified as White (21 [78%]). The primary language spoken was English (14 [82%]); only one family utilised a language interpreter. Most mothers had other live children (14 [82%]) and no known family history of CHD (12 [71%]). Initial diagnoses included a variety of cardiac anomalies, which varied in risk of mortality (Table 2).^39^

### Considerations and approaches prior to delivery

#### Accepting the diagnosis

Ultimately, all patients demonstrated understanding of the medical aspects of their child’s diagnosis and prognosis. While many shared their desire for a different reality, saying for example, “I wish my baby was [fine]” (8.M.1.L) or “I wish I knew when I was in the first trimester” (6.M.1.H), no one expressed or implied denial. Furthermore, they sought truth from providers, not false hope. One said, “I don’t need the sugarcoating. I just need to know how we think it’s going to go, and then I can be pleasantly surprised if it goes well” (10.F.2.H).

Families with diagnoses consistent with lower mortality risk said, “we’ve kind of accepted [it]” (8.M.1.L), and “however she comes into the world, we will love her.” (9.O.1.L). Families in the higher mortality risk group agreed, “we do know that there’s a 20% chance that he may not make it, but … what will be, will be” (1.O.2.H); and “after I came to the realisation that it was true … [we] had to accept it” (18.F.1.H).

#### Sense of control

When asked how much control they had, at least one from each family, of both higher and lower-mortality risk groups, discussed lack of control over their baby’s health: “It’s … out of my control. It’s … up to doctors and [my baby]” (18.M.1.H); “I wish I had more control over that stuff” (21.M.1.H); “There’s nothing I could do that would change or influence the outcome” (3.M.1.L).

Parents identified a few things they could control, such as “emotions” (11.M.1.L), “thoughts and attitude” and “self-care” (12.M.2.L), “gathering information” (5.F.2.H), and making personal medical decisions (20.M.1.H; 9.M.1.L). One explained, “the control we have is to stay involved and do the things we’re supposed to do” (8.F.1.L) because “I have no control over … how the baby’s going to be, but … [only] how we’re going to react, and what we’re going to do moving forward” (8.F.2.L).

#### Preparation and resources

The majority of patients felt well-prepared for their child’s birth saying they received “everything … needed to be prepared” (1.O.3.H), “I was as prepared as I could be” (10.F.3.H), and “as ready as I [could] be” (21.M.2.H). They “were so grateful . . . [to know they] were going to have a heart baby” (12.M.3.L), saying “to know up front is a lot better” (9.M.1.L) and they would “rather be prepared” (3.M.1.L). They appreciated “knowing the possibilities” (8.14.3.L) so “when something does happen, I can say, ‘okay, I was told about this’” (2.M.1.L).

Patients learned from repeated counseling by fetal cardiologists who “coached us . . . for several months before the birthing” (1.O.3.H). One mother said, “At the time, “I [didn’t] understand why they have to explain it over and over, but now that I look back . . . I’m glad they did because I understand it a lot more” (1.M.3.H). Others felt “overwhelmed after the first appointment,” (3.M.2.L) as “the first time you hear it . . . you’re not catching everything that could happen . . . but I got more in depth [information] this [second] time” (8.M.2.L).

Around two-thirds of patients who had received a diagnosis with a lower mortality risk sought out information online. They felt like it was “helpful” (3.M.2.L) to “know there are other people that you could talk [to]” (9.M.3.L) and “to see encouraging stories” (12.M.1.L). One said, “the Facebook group . . . has made me feel like way better . . . just knowing that there’s other families there” (11.M.3.L). In contrast, around two-thirds of patients facing higher-mortality risk “stay[ed] away from the Internet” (13.M.3.H). These respondents trusted the information they received from providers, saying, “You guys are going to tell me everything that I need to know . . . there’s no telling what you might find on the internet” (1.O.3.H), and “I’m not going to be able to get on the internet and pick out the answers . . . I rely on [doctors] to have information” (10.F.2.H). “I’m going to listen to what the doctors say . . . because I feel like I’m handling it so well. I don’t want to upset that balance” (5.M.1.H).

#### The role of hope

Regardless of the mortality risk of the diagnosis, families could simultaneously communicate possible death and hope for a positive outcome. While they were realistic about the future and their lack of control over many aspects of it, many families also “hope[d] for the best” outcome, whether probable or miraculous. “Even the doctor doesn’t know if she’ll make it . . . I’m hoping for good” (6.M.1.H).

Beside waiting for the future when the medical team would intervene, many parents believed, “nothing will help at this time other than God” (7.F.1.L). One mother explained she was “trusting” in “my doctors and in God” (4.M.1.L). A father agreed: “Divine spirit and . . . medical background, that could definitely help . . . ” (5.F.1.H). His wife said, “I’m just leaning on hope and faith at this point” (5.M.1.H), and later, “Even though things have gotten worse . . . if I get my healing miracle, I’ll get one, if not, I’ll deal with that when it comes” (5.M.2.H).

#### Emotional processing and coping

When asked how they are preparing for the future, nearly every participant in the lower-mortality risk group discussed childcare logistics, compared with only half of those in the higher-mortality risk group. Parents were planning for the “baby shower” (9.O.1.L) and “getting the nursery ready” (4.M.1.L), as well as maternity leave, “child care” (11.F.1.L), and the upcoming “added daycare cost” (8.F.1.L). One was grateful that “my in-laws are going to . . . be here and help out with our [older] daughter” (2.M.2.L). None of these families mentioned a possible bad outcome.

In contrast, three-quarters of families in the higher mortality risk group discussed their emotional preparation for a possible bad outcome when asked how they were preparing for the future. One said, “He’s going to be really sick” (1.O.1.H); “He’s either going to have multiple surgeries or a transplant . . . the sad part about [transplant] is that another baby has to die in order to give him a heart” (1.O.2.H). Others agreed, “It’s going to be hard . . . as the baby gets here” (20.O.1.H). “It’s going to be hectic” (10.M.2.H), “harder” (21.F.1.H) and “difficult . . . he’ll be in the ICU” (5.F.2.H). One said, “I can’t have surgery for him and then lose him at five months, that’ll kill me. I’d rather them just let him pass away on his own, right after birth than me lose him that late” (5.M.2.H).

The majority of patients in the higher mortality risk group also described dealing with life prenatally in discreet, manageable increments: “Take it day by day, and then as the time comes closer, you have to take it hour by hour, and don’t worry about tomorrow because today has enough worries” (21.M.3.H). Others discussed their commitment to “trying to be positive” (10.M.2.H) and “surround[ing] themselves [with] people that are not negative” (13.M.3.H) “to keep me positive . . . I refuse to mourn a baby still alive” (5.M.1.H). Some named distraction as an important coping mechanism – they tried to “stay busy” (10.M.1.H; 18.F.1.H), “to distract” themselves (13.M.3.H) “with other things, like work” (13.F.3.H), and to “not . . . think about it” (6.M.1.H; 21.F.1.H).

### Considerations and approaches after the child was born

Postnatally, as details about the diagnosis and prognosis crystalised, families found that their role evolved and the support they sought from providers often changed.

#### Communication

In contrast to their excellent prenatal preparation, many patients, regardless of their child’s condition, discussed how communication in the hospital *after* birth could be improved. One mother expressed confusion over the discharge plan, “Where are we going? What are we doing? Who do we talk to?” (8.M.3.L). She also “wish[ed] they would have done something” to improve communication among teams (8.M.3.L). Others desired dedicated time with a smaller group of providers, “not the entire rounds” (11.F.3.L). One said, “The only thing I would want changed was how the doctors addressed parents during rounds . . . Several times I had no idea what was going on . . . I often had questions that didn’t get answered” (20.M.3.H). Others agreed, “the terminology can become a little confusing” (10.F.3.H), “a lot of [it] I did not understand” (11.F.3.L).

Alternatively, when parents were familiar with the language and culture of the hospital, they seemed to feel more comfortable. Two mothers had prior children with CHD. One noted that having “previous knowledge . . . makes this experience better” because of clearer “expectations” about the upcoming steps and possibilities (21.M.2.H). While it was still “emotional” to be with her baby in the NICU, “the talks with the doctors and stuff, that wasn’t as scary the second time around” (21.M.3.H).

Regardless, parents felt reassured by the team spirit of providers who reportedly say “I’m on your side, we’re going to do this together” (21.M.3.H). They also valued providers’ creating physical and emotional space to feel supported (20.M.3.H) and to process new information.

#### Evolving role of parents – assuming control

After birth, some families assumed a new role on the team. Whereas prenatally, they felt their role was to “standby, pray, and wait” (1.M.2.H), to trust in providers and to prepare for the uncertain future, postpartum, they often became more actively involved in decision making, such as by consenting for procedures. Before giving birth, one mother said “I don’t know if I want that control” over “the decision on surgical intervention . . . I think I would feel guilty no matter what my decision was” (5.M.2.H). After birth, her posture changed. She advised future parents to be assertive, emphasising “not to feel like their opinion and their voice doesn’t matter . . . speak up” and “if a doctor says what you have to do, ask why . . . If you don’t know [what you want] . . . say you don’t know, and you need time” (5.M.3.H). She explained, “I felt everything was really rushed . . . I didn’t know what I had control over” (5.M.3.H). When asked specifically about their sense of control, others also expressed a desire to gain more control post-partum, when “it’s easier for me to wrap my mind around what we need to do” (3.M.3.L). One said, “This is my baby, I have to ask these questions, like, ‘why do we have to do this?’ . . . he was able to come off of [the nasogastric tube] a lot quicker because I asked that question” (21.M.3.H). As their role becomes more active, parents may require different communication and support from providers.

## Discussion

In this study, we found when families received a prenatal diagnosis for a child, they appreciated transparent, thorough information from providers. They understood the diagnosis and prognosis, and subsequently developed realistic worries and fears.^36^ To be sure, parents in this study longed for a healthy baby, but they communicated their understanding of the probability of that outcome through their word choice. For example, these parents consistently used the word “wish” to refer to a clearly impossible desire, such as to change an event that had occurred *in the past*, like the formation of their child’s heart, a delay in their knowledge of the malformation, or even their child’s death having occurred postpartum.

Yet, at the same time, families *hoped* for the best possible outcome for their child, and they seemed to cope with their new reality by focusing on that positive possibility, even if the probability of its occurring was minuscule. Physicians may misperceive hope as naïve optimism, potentially a negative adaptation, or as evidence of misunderstanding, for example, by asking questions about the distant future for a child with a poor prognosis. However, prior literature has demonstrated that parents can be hopeful while not optimistic that their child will recover,^40^ and even maintain hope for survival while understanding that their child will die.^41^ In this study, parents communicated acceptance around the high mortality risk, while often simultaneously relying on doctors and God, medicine, and faith as they “hope[d] for the best” – a “healing miracle” that may not come. This model of harmonious integration of prognostic awareness and hope has also been described in palliative care adult literature as an alternative to the historical understanding that hope can be unfounded or realistic, but not both at the same time.^42^

Through their hope, parents may actually demonstrate that they are informed and understand what is at stake death and suffering. As one father explained, “I am hopeful to have a fairly independent and capable child that would have a moderately long life expectancy,” and at the same time, “I don’t want my kid to suffer for five years and then die” (10.F.1.H). Put differently, these parents develop “critical hope,” which Dale Jacobs explains is “hope that allows us to imagine what is possible” and combines “a vision of the future toward which we can work with ‘the scientific analysis of reality.’”^43^ Thus, Jacobs explains, critical hoping is distinct from wishing and from “naïve optimism,” in that “we cannot just wish for something to happen, but must instead think reflexively about the situation and how we can assert our agency.”^43^

Thus, parents’ focus on positivity as a coping mechanism is a manifestation of “critical hope.” They understand the uncertainties and realities of their situation and acknowledge their lack of control over their child’s health. They assert the limited control they *do* have by granting trust to the medical team, by refusing “to mourn a baby still alive” (5.M.1.H), and instead, by focusing on positivity and their day-to-day lives. Parents may believe these actions will help them realise the best possible unfolding of events *in the future*. Families in this study, especially those who received diagnoses with a high risk of mortality, demonstrated their comprehension of the uncertainty of that envisioned future by also emotionally preparing for a possible bad outcome. Many did not engage in social media because they wanted to avoid despair or unfounded optimism in response to another parent’s story that may be not factual or may not apply to their own unique situation. Instead, they relied on information provided to them by medical providers so they could be “realistic.” These families truly valued thorough, honest information from providers and did not feel it took away their hope.

Acknowledging the presence of conflicting emotions within hope, optimism, wish, and realism may help to identify interventions to better support positive parental adaptation. Certainly, some families use these terms in different ways, misunderstand the prognosis, and hold naïve optimism about the future. Future interventions may focus on helping providers to understand individual parents’ evolving conceptualisation of these key concepts in relation to their understanding of their child’s diagnosis. Ultimately, teasing out the meaning behind individuals’ communicated hopes may enhance effective communication.

In prior analysis of this data, we found that the experience of parents prenatally was defined by uncertainty around both concrete questions and long-term unknowns. Receiving answers to concrete questions about appointments, logistics, and future steps helped parents gain some sense of control.^36^ Our findings in this study support that earlier observation. Through their coping strategies, families sought out a sense of agency. Some, especially those who received a diagnosis with a lower risk of mortality, felt empowered by seeking out information online and advice from other parents. As has been described previously of parents of children with developmental disabilities,^44^ families in our study likely achieved a sense of agency by reducing their sense of uncertainty. We found here that parents’ coping strategies evolved prenatally to postnatally: parents moved from feeling helpless to more in control as the prenatal diagnoses became clearer. When parents met their child face-to-face after birth, and prognostic uncertainty diminished, their sense of and desire for control often expanded. They felt more comfortable questioning providers and actively participating in generating care plans. Providers’ communication may need to change as well, both to help parents to cope adaptively with postnatal events and to encourage more effective participation in decision making in their child’s care.

### Considerations and limitations

The purpose of this study was to describe families’ experiences from prenatal diagnosis until after birth. Future studies conducted at multiple institutions with a greater diversity of families can further inform knowledge on this important topic. Most mothers interviewed in this study had already had a successful pregnancy. The experience of women who do not have prior children may be different, with distinctive coping mechanisms. Future research should investigate how these attributes affect emotional processing.

Additionally, interviewees in this study may have perceived the interviewer (KWH) as part of the medical team despite measures to mitigate this possibility, which could have influenced participant responses. Even if they understood her role as a researcher, engaging in the study interviews may have served as a coping mechanism for patients, which could itself be studied as an intervention in the future.^45^

## Conclusion

This study suggests that parents’ journey following a prenatal diagnosis of CHD is psychologically complex, affected in important ways by the child’s prognosis, by the inevitable waiting, and by the child’s ultimate birth. Prior to delivery, parents typically seek to maintain hope as they work to understand their child’s diagnosis. However, their coping styles and approaches to information gathering appear to vary depending on the severity of their child’s prognosis. Expectant parents whose child has a higher risk of mortality seem to demonstrate “critical hope” by focusing on positivity and their day-to-day life as a coping mechanism. They also tend to rely more exclusively on clinician-provided information, as opposed to that found on the internet. Regardless of prognosis, parents’ sense of control often expands following delivery, leading them to seek more active engagement in clinical decision making. Understanding the trajectories of parental emotional processing and coping after prenatal diagnosis of CHD, including influencing factors like prognosis severity, may help clinicians meet families’ needs more effectively.

## Supplementary Material

Supplement

## Figures and Tables

**Table 1.** Demographic characteristics^a^

	Characteristic	No.	%
**Participant Information**	**Role**		
	Mother	16	59
	Father	8	30
	Grandmother	1	4
	Great-Grandmother	1	4
	Friend	1	4
	**Race/Ethnicity**		
	White (European)	20	74
	White (Middle Eastern)	1	4
	Black / African-American	3	11
	Hispanic / Latino	3	11
	**Age, median (IQR), years**		
	Of mother	30 (27.3–34.8)	
	Of family member / support person	30 (26–42)^b^	
	**Occupation**		
	Medical field (caregiver, healthcare administrator, healthcare coder, health systems worker, home health assistant, nurse, physiologist, imaging technician)	9	33
	Business / Sales	8	30
	Homemaker	6	22
	Mechanic / Electrician / Construction	2	7
	Lawyer	1	4
	Firefighter	1	4

	**TOTAL**	27	100

**Family Information**	**Distance of mother from hospital, median (IQR), miles**	32 (14.5–92)	
	**Gestation at first consult visit, median (IQR), weeks**	25 (21–32)	
	**Primary Language**		
	English	14	82
	Spanish	1	6
	Arabic	1	6
	Other (African Dialect)	1	6
	**Gravidity of mother**		
	1	3	18
	2	6	35
	3	1	6
	4	4	24
	5	2	12
	7	1	6
	**Parity of mother**		
	0	3	18
	1	6	35
	2	5	29
	3	2	12
	4	1	6
	**Prior miscarriages**		
	0	12	71
	1	3	18
	2	2	12
	**Significant Family History of CHD**		
	No	12	71
	Yes	5	29
	**Fetal diagnosis**		
	Pulmonary Stenosis/Atresia	3	18
	Coarctation of the Aorta	3	18
	Tetralogy of Fallot	2	12
	Ebstein Anomaly	2	12
	Transposition of the Great Arteries	2	12
	Hypoplastic Left Heart Syndrome	2	12
	Hypoplastic Right Heart Syndrome	1	6
	Dilation of the Aorta	1	6
	Atrioventricular Septal Defect	1	6
	**Current baby vital status post-partum**		
	Alive	12	76
	Deceased	5	24
		
	**TOTAL**	17	100

Abbreviation: IQR, interquartile range.

a Table originally printed in Harris KW, Brelsford KM, Kavanaugh-McHugh A, Clayton EW. Uncertainty of Prenatally Diagnosed Congenital Heart Disease: A Qualitative Study. *JAMA Netw Open.* 2020.

b Three missing data points, as ages of two fathers and one great-grandmother unknown.

**Table 2.** Diagnoses included in this study grouped by mortality risk.

Lower Risk of Mortality Score of 5 or less^a^		Higher Risk of Mortality Score of 6 or greater^a^	
Diagnosis	Family Number	Diagnosis	Family Number
Coarctation of the aorta	12, 15	Severe Ebstein’s Anomaly	10*
Tetralogy of Fallot	8, 11	Hypoplastic Left Heart Syndrome	1, 18*
Dilation of the Aorta	3	Hypoplastic Right Heart Syndrome	5*, 13
Simple Transposition of the Great Arteries	2	Complex Transposition of the Great Arteries	21
Pulmonary Stenosis	4, 7	Pulmonary Atresia with other complications	6*
Atrioventricular Septal Defect	9	Double outlet right ventricle, coarctation of the aorta, congenital diaphragmatic hernia	20*

a Allan LD, Huggon IC. Counselling following a diagnosis of congenital heart disease. *Prenatal diagnosis*. 2004;24(13):1136–1142.

* Baby died after birth.

**Table 3.** Thematic analysis summary of illustrative quotes

Grouping	Theme	Sub-theme (if applicable)	Illustrative Quotes^a^
Prenatal Considerations and Approaches	Accepting the Diagnosis		“I don’t need the sugarcoating. I just need to know how we think it’s going to go, and then I can be pleasantly surprised if it goes well” (10.F.2.H)
			“We do know that there’s a 20% chance that he may not make it, but like she [my daughter] says, what will be, will be” (1.O.2.H)
	Sense of Control		“I don’t feel like it’s something I can control. He’s growing the way he’s growing, the surgery’s going to happen the way it happens, the birth is going to happen the way it happens. I don’t feel like there’s anything I can really do to control it other than just having the information at hand and knowing how to mentally prepare for anything” (2.M.1.L)
	Preparation and Resources	Well-Prepared	“I think for the most part [we were] as well as you can be prepared. We were so grateful that actually we knew we were going to have a heart baby… I cannot imagine having gone in to have a baby and then finding all that out.” (12.M.3.L)
		Avoiding the Internet	“I’m not going to [go online] because at this point I’m just going to listen to what the doctors say and then just keep doing what I’m doing because I feel like I’m handling it so well. I don’t want to do anything to upset that balance.” (5.M.1.H)
		Seeking Information Online	“The Facebook group… has made me feel like way better about like everything. That community is very tight knit it seems in offering support. And not that we… Not that I’ve even asked, it’s just like knowing that there’s other families there. So, I think that resource list was really helpful early on.” (11.M.3.L)
	Role of Hope		“When they told me that she has this bad disease, I was like heart-broken … Even the doctor doesn’t know if she’ll make it or not… I’m just hoping for good, I’m wishing for good” (6.M.1.H)
	Emotional Processing and Coping	Day-to-Day	“Take it day by day, and then as the time comes closer, you have to take it hour by hour, and don’t worry about tomorrow because today has enough worries” (21.M.3.H)
		Positivity	“I feel like it’s a worse-case scenario than it was before, but I’m hoping she’ll grow and things get better. I’m still trying to be positive about it” (10.M.2.H)
			“I just feel like I just got to be strong for my family. If I let myself down, then I pretty much let everybody else down so I’ve got to stay positive. I keep everything upbeat and just make it less stressful and less sad with the situation.” (13.F.1.H)
		Distraction	“It’s good to think about it every once in a while, but I try not to stay thinking about it. I try to distract myself from just thinking about that one thing… I’m always about to start cooking. I just whistle things here. I read to my kid, I cook, I go for walks, go do stuff to distract myself” (13.M.3.H)
		Preparing for Bad Outcome	“I can’t have surgery for him and then lose him at five months, that’ll kill me. I’d rather them just let him pass away, on his own, right after birth than me lose him that late” (5.M.2.H)
		Practical Logistics	“Just kind of the standard things that you would do for a baby [I am doing to prepare]. Getting the nursery ready, get everything washed, you know, get in the house, kind of baby prepped.” (4.M.1.L)
Postpartum Considerations and Approaches	Communication	Room for Improvement	“The only thing I would want changed was how the doctors addressed parents during rounds. I was there literally every day. Several times I had no idea what was going on … I often had questions that didn’t get answered” (20.M.3.L).
		Improved understanding from prior experience	“I think that the NIC unit was very the same, and it was just kind of a waiting game. It was just emotional in the aspect of like you see your baby, there’s too many wires and all that stuff, that was the only emotional part about it, but not the words. The words, like then the talks with the doctors and stuff, that wasn’t as scary the second time around.” (21.M.3.H)
	Evolving role of parents - Assuming Control		“It’s easier for me to wrap my mind around what we need to do. Because there just aren’t [as many] outstanding issues” (3.M.3.L)
			“This is my baby, I have to ask these questions, like why do we have to do this?” (21.M.3.H)

a Participant quotes are identified first by family number (1–21), then by participant role (M = mother, F = father, O = any other support person), then by time point of interview (1–3), and finally by risk of mortality (H = higher, L = lower). Thus, 5.F.2.H indicates a quote from a father of family number 5 during the second interview with a higher risk of mortality diagnosis.
